# Post-traumatic stress disorder in French Guiana: prevalence and risk factors in the general population

**DOI:** 10.3389/fpubh.2025.1668105

**Published:** 2025-11-26

**Authors:** Mathieu Nacher, Astrid Van Melle, Estelle Thomas, Johanna Pavie, Blandine Solignat, Imane Benradia, Deborah Sebbane, Eva Aernout, Jean-Luc Roelandt, Caroline Janvier, François Lair, Vincent Bobillier

**Affiliations:** 1 CIC INSERM 1424, CHU Guyane, Cayenne, French Guiana; 2 UA17 INSERM Amazonian Population Health, University of French Guiana, Cayenne, French Guiana; 3 Plateforme Rétablissement, Groupe SOS, Cayenne, French Guiana; 4 CHU Guyane, Cayenne, French Guiana; 5 Centre collaborateur de l'OMS pour la recherche et la formation en santé mentale – CCOMS, Service de l'EPSM Lille-Métropole, Lille, France; 6 Service de psychiatrie, Centre Hospitalier de Cayenne, Cayenne, French Guiana

**Keywords:** post traumatic stress disorder, migration, age, gender, French Guiana

## Abstract

**Background:**

The context of French Guiana—characterized by frequent violence and intense immigration from regions where traumatic events are common—may lead to a different epidemiology of post-traumatic stress disorder (PTSD) compared with mainland France. In the absence of prior data, we aimed to estimate the overall prevalence of PTSD in the general population of French Guiana, along with its prevalence across different sociodemographic groups.

**Methods:**

A cross-sectional survey was conducted between March and August 2021, interviewing 881 subjects aged >17 years.

**Results:**

The overall prevalence of PTSD was 7.6 [95%CI = 5.9–9.5]. After adjusting for potential confounders among sociodemographic variables, only female sex (adjusted prevalence ratio (aPR) = 2.1, 95%CI = 1.2–3.6, *p* = 0.006), being a student (aPR = 2.4, 95%CI = 1.0–5.6, *p* = 0.05), being from Suriname (aPR = 5.8, 95%CI = 1.0–33.4, p = 0.05), not having completed primary education (aPR = 3.4, 95%CI = 1.3–8.9, *p* = 0.01), and being separated or divorced (aPR = 3, 95%CI = 1.1–8.2, *p* = 0.02) were significantly associated with PTSD. Of the 67 individuals with PTSD (46.3%), 31 sought care, 21 (31.3%) reported having received treatment for their symptoms, and 29 (43.3%) reported using alternative treatments, mostly plant-based traditional medicine. Of the 394 individuals who had experienced an unusual traumatic event, 67 (17%) developed PTSD.

**Conclusion:**

We found that the prevalence of PTSD is approximately 10 times higher than in mainland France and that substantial differences exist across origin, gender, and age group. We also observed that the majority of individuals do not seek treatment for their symptoms, and those who do often combine Western and traditional medicine. These findings emphasize the need for culturally sensitive approaches to screening and care.

## Introduction

French Guiana is a South American French overseas territory located between Brazil and Suriname. It has the highest gross national product (GNP) per capita in Latin America, a promise of riches that has attracted many immigrants from Latin America and the Caribbean, who are fleeing socioeconomic hardships, natural disasters, or violence in their countries. As a result, a third of the population, and nearly half of adults, are of foreign origin. Immigration is mostly regional from surrounding countries, mainly Suriname, Brazil, and Haiti. Immigration waves have affected different regions at different times. The ethnic makeup of French Guiana is hence a mix of Creole, Maroon, Amerindian, Caucasian, Chinese, Hmong, and many other ethnic groups. Socioeconomic differences across ethnic groups are not trivial, but ethnic statistics are not allowed in France, so it is not easy to ascertain how important these differences are. The country of birth, however, often correlates with socioeconomic difficulties, and thus, being foreign is often used as a proxy for poverty.

French Guiana has the same social protections, health system, and educational system as mainland France, but half of its population lives below the poverty threshold. Another singular sociodemographic feature of the territory is its youth, with a median age of 24 years. French Guiana also has the highest birth rate in Latin America.

Traumatic events, such as war, violence, and natural disasters (1–3), are known causes of post-traumatic stress disorder (PTSD). The prevalence of PTSD is high in high-income countries, with significant differences between sociodemographic groups (4). Women and younger age groups have a higher prevalence of PTSD (1). However, global studies including low- and middle-income countries allow for the comparison of the distribution of trauma and PTSD across the world. Different sociopolitical environments impact the distribution of traumatic events. Traumatic event prevalence rates are thus higher in countries emerging from conflict, but the distribution of traumatic event types varies between regions. Traumatic events are often the reason many immigrants leave their countries and arrive in French Guiana. Furthermore, during the migration process, some may be subjected to violence, including sexual violence against women (5, 6).

There are thus significant social contrasts that are difficult to capture in global statistics: French Guiana is a mix of France and shantytowns reminiscent of low- and middle-income countries, with individuals who have very different life trajectories, notably regarding their exposure to various traumatic events. Within French Guiana, violent events are also prevalent, with the highest incarceration and homicide rates in France. A previous study showed that nearly 3 out of 4 inmates had a psychiatric condition, and the prevalence of PTSD among inmates was 15% (7).

French Guiana thus represents a post-colonial frontier where migration, socioeconomic exclusion, and historical inequities converge, creating conditions that amplify both exposure to traumatic events and barriers to care. Understanding PTSD in this setting, therefore, extends beyond psychiatry into social epidemiology. We hypothesized that the context of French Guiana—frequent violence and intense immigration of individuals from areas where traumatic events are common—may lead to a different epidemiology of PTSD relative to mainland France. Given the heterogeneous makeup of the population of French Guiana and the dearth of data on various psychiatric conditions, we aimed to estimate the overall prevalence of PTSD and its prevalence in different sociodemographic groups.

## Methods

### Mental health in the general population survey

This study analyzed the cross-sectional Mental Health in the General Population (MHGP) survey, which was first implemented in 1999 by the French WHO Collaborating Center (WHOCC). The MHGP survey was administered at various sites in mainland France and its overseas territories, including, for the first time, French Guiana. The goal was to recruit 900 individuals at each site, with inclusion criteria comprising: (i) proficiency in French, (ii) provision of informed consent, (iii) age over 18 years, and (iv) not being a resident of a care institution. Subjects were selected through a quota-sampling method to obtain a representative sample of the general population of each site concerning age, gender, educational level, and occupation, according to census figures from 1999 provided by the French National Institute of Statistics and Economic Studies (INSEE).

### Study sample

The survey “Mental Health in the General Population: Images and Realities” (MHGP-IR) was conducted in French Guiana across six municipalities—Cayenne, Macouria, Matoury, Roura, Montsinéry-Tonnégrande, and Rémire-Montjoly—between March and September 2021. A total of 900 individuals aged 18 years and older were interviewed. Data were collected anonymously by trained interviewers using questionnaires administered through face-to-face interviews conducted at various locations, including on the streets, in order to increase representativeness. Questionnaires were available in four different languages, with specific versions in French, Portuguese, and English. For French Guianese Creole, surveyors translated the French version of the questionnaire. Quota sampling was applied based on the 2017 population census data, considering age, gender, education level, and socio-professional group.

### Data used

For each subject, we used the Mini-International Neuropsychiatric Interview (MINI) (French version 5.0.0), a standardized psychiatric interview based on the DSM-IV/ICD-10, to screen for mental health disorders. The MINI’s inter-rater and test–retest reliability and validity have been previously verified (Lecrubier, 1997). The MINI is based on the criteria outlined in the 10th edition of the International Classification of Diseases (ICD-10) and in the fourth edition of the Diagnostic and Statistical Manual of Mental Disorders (DSM-IV). All MHGP interviewers (nurses and psychologists) were trained by WHOCC experts to administer the MINI. The present study only addressed current mental health problems, notably post-traumatic stress disorder (F43.1 in the ICD-10). Current PTSD is defined as reporting ongoing symptoms at the time of the survey. The surveyed population was asked if they had been through an unusual traumatic experience (war, a natural disaster, contact with death or violence, etc.). The list of possible responses was largely fixed and included the following: an earthquake, witnessing a serious accident, being the victim of an attempted rape, the sudden death of someone in the respondent’s circle, a flood or deluge, taking part in combat, being held hostage, war, a terrorist attack, killing someone, a kidnapping, a natural disaster, a fire, being the victim of an assault, discovering a corpse, and other events.

We used other variables known to be associated with mental health issues: sex, age (≤25, 25–35, 35–45, 46–55, 56–65, and >65 years), education level (no education or primary level, secondary level, or university level), marital status (married or cohabiting with a partner, single, separated, divorced, or widowed), occupation (employed, unemployed or inactive, i.e., retiree, student, or stay-at-home parent), and categorized monthly household income.

### Statistical analysis

Overall, 881 participants were analyzed.

We first computed the overall prevalence and 95% confidence intervals [95%CI] for PTSD and then estimated stratum-specific rates for different sociodemographic categories. Cross-tabulations using chi-squared tests looked for statistical differences.

Finally, we performed simple and multivariate analyses to compute crude and adjusted prevalence ratios using Poisson regression. Poisson regression with robust variance was used to directly estimate prevalence ratios, avoiding the overestimation associated with odds ratios in cross-sectional studies. The models were adjusted for factors potentially associated with the prevalence of PTSD, including other mental health diagnoses, site, age, sex, marital status, education level, occupational activity, and monthly income. To avoid overfitting, we computed a sociodemographic model and a model for other psychiatric diagnoses. Hosmer–Lemeshow goodness-of-fit tests were used to assess model fit. Prevalence ratios (PR) and 95%CI were computed. Statistical significance was set at 5%.

To visually display the relationship between PTSD and other mental health issues, a heatmap was plotted. The proportion of individuals who had experienced a traumatic event was calculated, the most frequent events were plotted, and a word cloud was created. Data analysis was performed using STATA 16 (Stata Corporation, College Station, Texas, USA).

### Ethical aspects

The protocol of the Santé Mentale en Population Générale (SMPG) survey was declared and approved by an Ethical Committee (#CCTIRS 98.127) prior to the survey. All the participants provided informed written consent to the survey and the publication of its results prior to their participation.

## Results

### Sociodemographic characteristics

Table 1 shows the sociodemographic characteristics of the sample. Two-thirds of the sample population were of French origin. More than a third of the sample had less than a secondary education, and 14.4% had a monthly family income below €840. Fewer than half of the sample were couples. Approximately two-thirds of the non-students were professionally active.

**Table 1:** Sociodemographic characteristics of the sample, SMPG study.

	*N*	%
Origin		
Brazil	50	5.68
Mainland France	139	15.80
Guyana	15	1.70
French Guiana	382	43.41
Haiti	173	19.66
French Antilles	62	7.05
Suriname	20	2.27
Other	39	4.43
Education		
Less than primary	78	8.85
Less than secondary	270	30.65
Completed secondary	533	60.50
Activity		
Active	505	57.32
Inactive	282	32.01
Student	94	10.67
Monthly family income		
< €534	73	8.29
€534 to 840	54	6.13
€840 to 1,300	88	9.99
€1,300 to 2,520	202	22.93
€2,520 to 6,410	189	21.45
> €6,410	29	3.29
Does not wish to answer	246	27.92
Marital status		
Married or cohabiting	376	42.68
Single	402	45.63
Separated or divorced	56	6.36
Widowed	30	3.41
Does not wish to answer	17	1.93
Age group (years)		
<26	177	20.09
26–35	199	22.59
36–45	157	17.82
46–55	146	16.57
56–65	128	14.53
>65	74	8.40

### Prevalence of PTSD

Table 2 shows the prevalence of PTSD in the sample and in different subgroups. The global prevalence was 7.6% [95%CI = 5.9–9.5], but the results varied widely between groups. Women, respondents from Suriname and Haiti, those under 26 years of age, individuals with an education level less than a primary education, individuals with a family income lower than €840, and students had higher prevalence rates of PTSD. After adjusting for potential confounding among sociodemographic variables, only female sex (adjusted prevalence ratio (aPR) = 2.1, 95%CI = 1.2–3.6, *p* = 0.006), being a student (aPR = 2.4, 95%CI = 1.0–5.6, *p* = 0.05), being from Suriname (aPR = 5.8, 95%CI = 1.0–33.4, *p* = 0.05), not having completed primary education (aPR = 3.4, 95%CI = 1.3–8.9, *p* = 0.01), and being separated or divorced (aPR = 3, 95%CI = 1.1–8.2, *p* = 0.02) were significantly associated with PTSD.

### Care and treatment

Of the 67 subjects with PTSD, only 31 (46.3%) sought care: 17 (25.4%) consulted a general practitioner or nurse, and 20 (29.8%) consulted a psychiatrist or psychologist. Cross-tabulations of medical or traditional treatment-seeking behavior showed no significant differences in treatment seeking by gender, income, activity, or education. The cross-tabulation of health-seeking behavior and country of origin showed a statistically significant difference, with those from Haiti appearing to be less likely to seek treatment. Overall, of the 67 participants, 21 (31.3%) declared having had treatment for their symptoms, and 29 (43.3%) took alternative treatments, mostly plant-based traditional medicine. Those who received medical treatment were also more likely to take alternative treatments than those who did not take medical treatment (15 out of 29 vs. 6 out of 38, *p* = 0.002).

### Proportion reporting a history of the traumatic event

**Figure 1:**
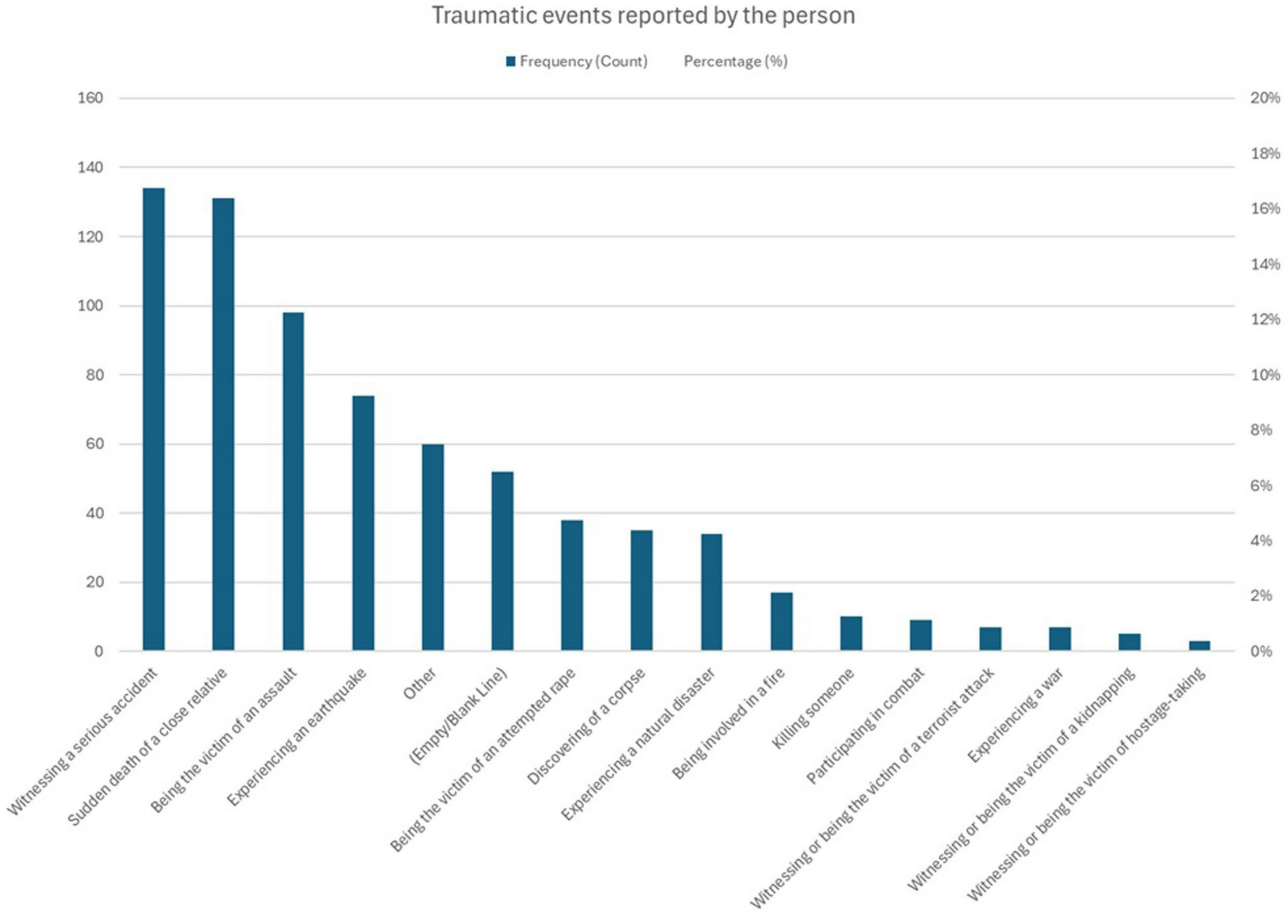
Traumatic events.

Overall, of the 881 participants, 394 (44.7%) answered that they had experienced an unusual traumatic event and 139 thought frequently or dreamed about it or had the impression of reliving it. Of the 394 individuals who had experienced an unusual traumatic event, 67 (17%) developed PTSD. The nature of the most common events is shown in Figure 1. When broken down by country of origin, individuals from Haiti most frequently reported experiencing an earthquake (Supplementary Figure S1). Individuals with low family income reported more violent assaults (Supplementary Figure S2). Supplementary Figures S3, S4 show PTSD prevalence by age group and by sex, respectively (see Table 2).

**Table 2:** Global and group-specific prevalence of PTSD in French Guiana, SMPG study, 2021.

Variable	*N*	PTSD prevalence (%) (95%CI)
Global	881	7.6 (5.9–9.5)
By origin		
Brazilian	50	4 (0.5–13.7)
Mainland France	139	4.3 (1.6–9.1)
Guyana	15	6.6 (0.1–31.9)
French Guiana	382	6.8 (4.5–9.8)
Haiti	173	11 (6.7–16.6)
Antilles	62	1.6 (0.0–8.6)
Suriname	20	2 (0.5–43.3)
Other	39	20.5 (9.2–96.4)
Education level		
Less than primary	78	11.5 (5.4–20.7)
Less than secondary	270	4.4 (2.3–7.6)
Completed secondary	533	8.6 (6.3–11.3)
By activity		
Student	94	19.1 (11.7–28.5)
Active	505	6.1 (4.2–8.6)
Inactive	282	6.3 (3.8–9.9)
By age group (years)		
<26	177	11.8 (7.4–17.5)
26–35	199	10 (6.2–15)
36–45	157	5 (2.2–9.8)
46–55	146	7.5 (0.4–13.1)
56–65	128	3.9 (1.2–8.8)
>65	74	2.7 (0.3–9.4)
Sex		
Female	464	10.1 (7.5–13.2)
Male	417	4.8 (2.9–7.3)
Monthly family income (euros)		
<840	127	11.8 (6.7–18.7)
840–2,520	290	5.5 (3.1–8.8)
>2,521	218	6.8 (3.9–11)
Does not wish to disclose	246	8.5 (5.3–12.7)
Marital status		
Married or cohabiting	376	6.3 (4.1–9.3)
Single	402	8.7 (6.1–11.9)
Separated or divorced	56	10.7 (4–21.8)
Widowed	30	3.3 (0.0–17.2)

### Relationship of PTSD with other psychiatric diagnoses

**Figure 2:**
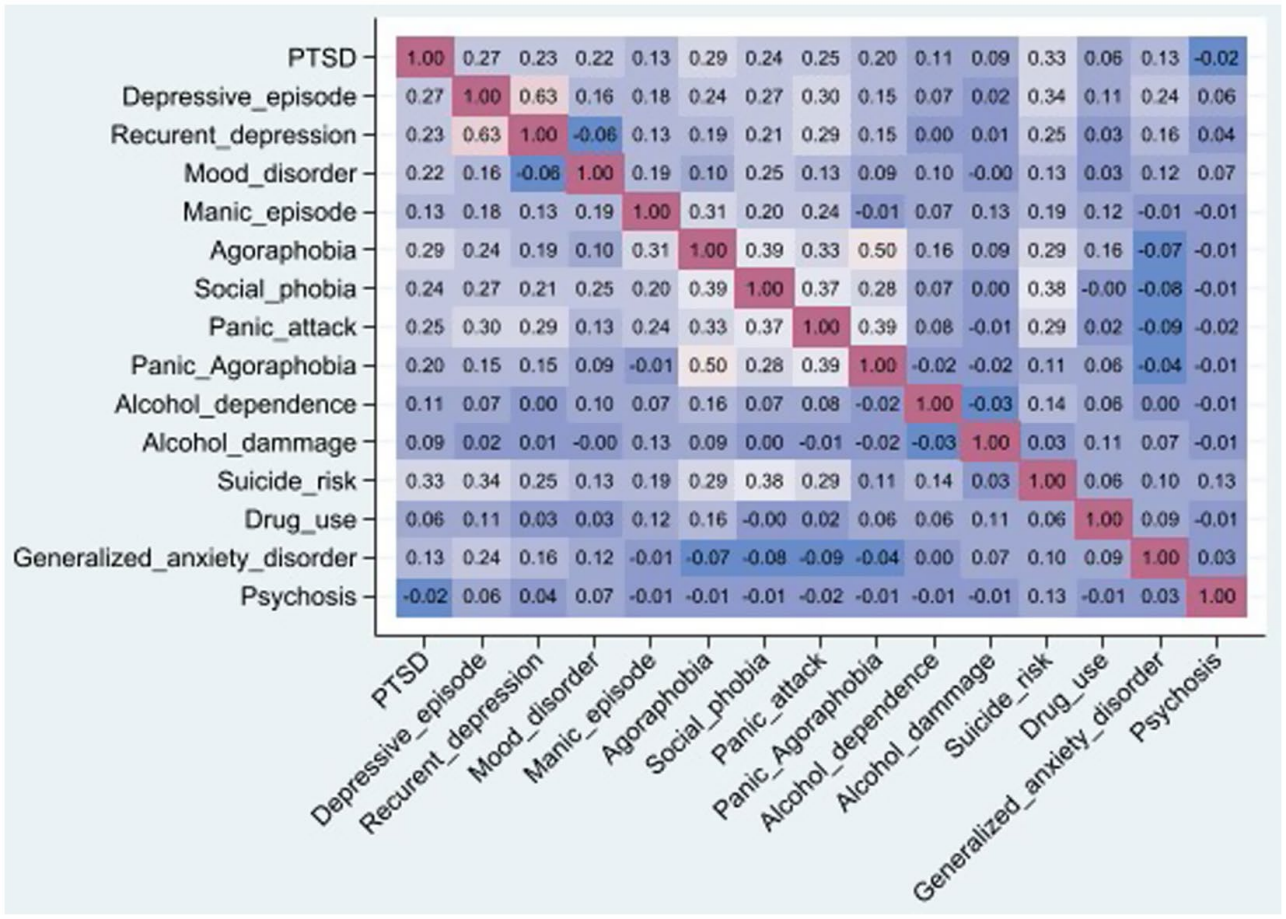
Heatmap of the associations between different psychiatric diagnoses, SMPG 2021, French Guiana.

Figure 2 shows the heatmap of different psychiatric diagnoses. PTSD was most related to suicide risk, depressive episodes and recurrent depression, mood disorder, agoraphobia, social phobia, panic, and psychosis.

**Figure 3:**
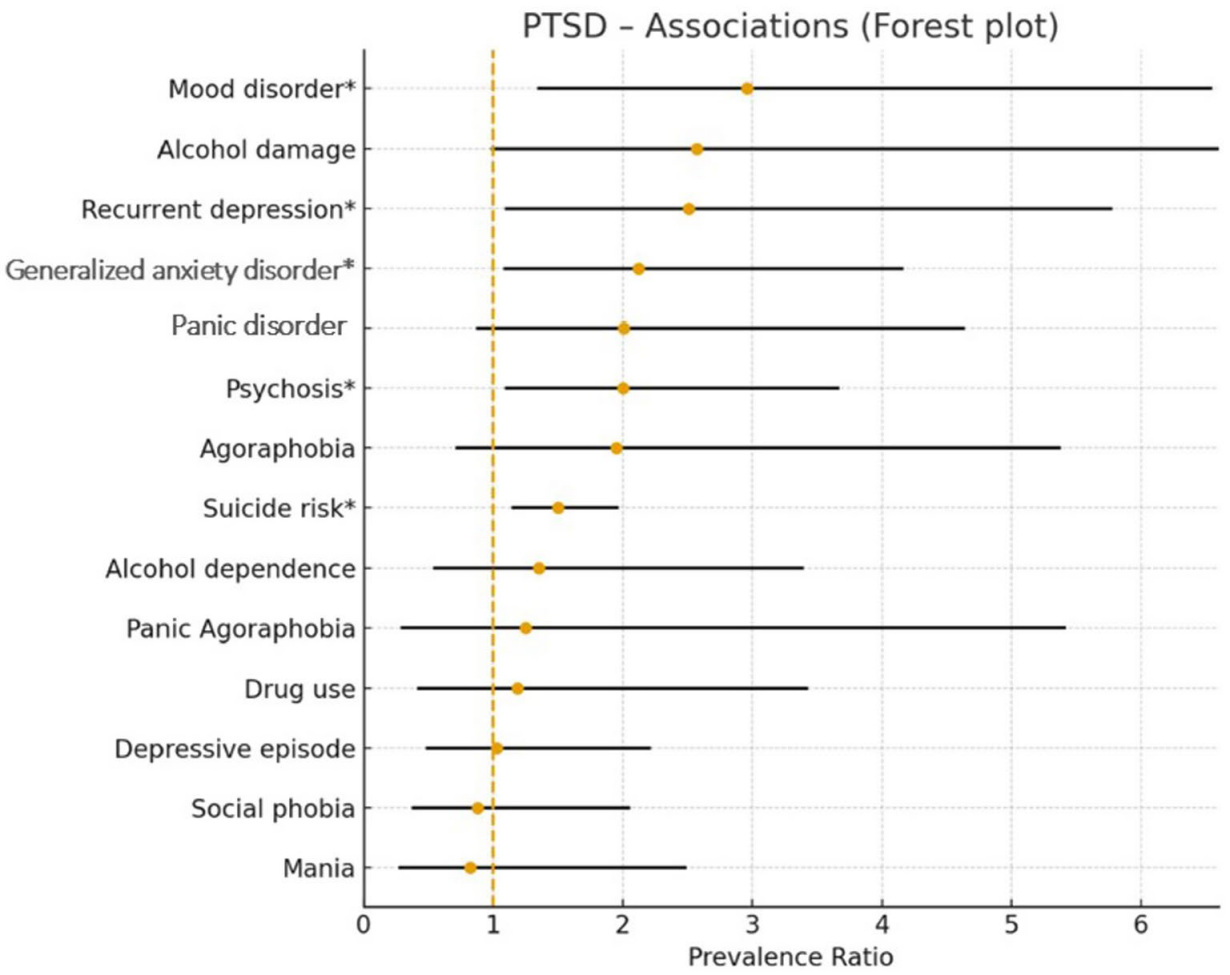
Adjusted prevalence ratios between PTSD and different psychiatric disorders, SMPG 2021, French Guiana.

Figure 3 shows the forest plot representing the adjusted relationship between PTSD and different psychiatric diagnoses. After adjustments, suicide risk, alcohol damage, recurrent depression, mood disorder, generalized anxiety disorder, and psychosis were independently associated with PTSD.

## Discussion

In this study, we reveal that the prevalence of PTSD in a carefully selected sample of the general population of French Guiana was 7.6% [95%CI = 5.9–9.5]. The bivariate analysis showed that a number of sociodemographic variables were associated with statistically significant differences. As shown elsewhere, female participants were twice as likely to report PTSD as male participants, 10.1% vs. 4.8%, respectively. There was a negative linear trend between age and PTSD prevalence, with younger age groups having greater prevalence rates than older age groups. French-born (mainland France or its overseas territories) had lower prevalences than those born in Haiti and Suriname. Students had a far greater prevalence than professionally active and inactive individuals, 19.1% vs. 6.1 and 6.3%, respectively. Having a monthly family income below €840 appeared to be associated with a higher prevalence, but this was not statistically significant. Marital status was not significantly associated with any differences in PTSD prevalence. The multivariate sociodemographic model only found female sex, not having completed primary education, being divorced or separated, being from Suriname, and being a student as significantly independent associated variables. The multivariate model, including other psychiatric diagnoses, found that suicide risk, alcohol damage, recurrent depression, mood disorders, generalized anxiety, and psychosis were independently associated with PTSD. Fewer than half of those with PTSD sought care for their symptoms, and 31.3% received treatment, which is lower than global estimates and, more specifically, estimates from high-income countries (48.8 and 53.5%, respectively) (8). Among those with PTSD, 43.3% used alternative medicine, and those who did were also more likely to use medical treatment, demonstrating that individuals often rely on both practices to manage their symptoms and that the usual dichotomy between Western and traditional medicine fails to capture actual patient treatment-seeking behavior. Patients perceive clear advantages and disadvantages to biomedical and traditional care in medically pluralistic settings. Healthcare provider, healthcare system, and peer-level factors combine to influence patients’ therapeutic itineraries (9). Those with higher incomes and women were more likely to consult for their symptoms than other groups.

The proportion of individuals reporting a traumatic event was lower than that reported in the MHGP study in France or estimates in the Americas (44.7% vs. 72.7 and 60%, respectively) (8, 10). This is counterintuitive and appeared to be at odds with our initial hypothesis that, in French Guiana, the probability of encountering traumatizing events was higher than in France, and that among immigrants, the probability that they had traumatic events in their country of origin was even higher. Studies in a range of countries also yield a proportion of approximately 70% of the population reporting traumatic events, but with wide variations, suggesting cultural differences in addition to sociopolitical circumstances. For instance, in Iraq, a country that has suffered multiple hardships, the proportion was 56%, which is much lower than in the United States (82.7%). Perhaps, and this is speculative, some highly exposed populations eventually develop greater resilience to traumatic events.

PTSD prevalence rates in the general population vary widely across different countries. In French Guiana, it appeared substantially higher than in other high-income countries. In mainland France, the SMPG study, using the same design with a sample of 36,000 adults, found a prevalence of 0.7%. In the USA, the prevalence of post-traumatic stress disorder was 1% in the total population, 3.5% among civilians exposed to physical attacks, 4% among Vietnam veterans who had not been wounded, and 20% among veterans wounded in Vietnam. In Germany, PTSD prevalence was 1% in male individuals and 2.2% in female individuals. Traumatic events and PTSD are associated with all other mental disorders. The prevalence among Haitian immigrants in French Guiana was slightly higher than among Haitian immigrants in Southern Brazil, where it was estimated at 9.1% in a smaller sample (11). The prevalence in French Guiana, however, appears to be lower than in low- and middle-income countries where information is available. A meta-analysis and systematic review in sub-Saharan Africa found an overall pooled prevalence across all studies of 22% [95%CI = 13–32%] with wide variations across the individual studies, ranging from 2% [95%CI = 2–2%] to 74% [95%CI = 72–76%] (12). Prevalence estimates differed substantially across regions by population-level exposure to war or armed conflict. The WHO estimates from a systematic review and meta-analysis in conflict settings found a 15·3% prevalence of PTSD [95%CI = 9.9–23.5%] (13). Finally, the overall prevalence in the general population of French Guiana was lower than the 15% found among inmates at French Guiana’s sole correctional facility (7). This is not surprising and has been described elsewhere (14).

Although different sociodemographic factors are associated with traumatic event exposure and PTSD in high-income settings, it is not always the case in low-income and post-conflict societies (1, 12). The risk factors in high-income contexts reflect existing knowledge of risk factors for PTSD, but the high frequency of exposure to traumatic events in all sociodemographic groups in low-income or violence-ridden countries blurs these associations. In French Guiana, the mix of being a European territory combined with local poverty and violence, and of individuals whose life trajectories to reach French Guiana were marked by traumatic events, makes the situation somewhat intermediate between OECD countries and low- and middle-income countries.

The present study has a number of limitations. Although the MINI is the most common tool for assessing mental health issues, its psychometric reliability rests on the surveyor’s training and on the interviewee’s declaration of symptoms. The version used was based on the DSM-IV, which may lead to an overestimation of the prevalence of PTSD. However, the difference in PTSD prevalence is generally small, and given the magnitude of differences between French Guiana and France, or between subgroups, we are confident about our results. A further limitation is that the MINI evaluates the presence or absence of symptoms but not how severe they are. The MINI questionnaire may not be optimal for individuals from the very different culture of French Guiana, and translation by interpreters may introduce biases. A social desirability bias in a small, socially interconnected community could have led to underreporting of psychiatric symptoms or traumas. Finally, other studies may use different tools (15), making prevalence comparisons difficult to interpret. The prevalence of PTSD broken down into different subgroups leads to statistical power loss and large confidence intervals when the subgroups are small. Moreover, the cross-sectional design misses evolving trends in a context of unfolding potentially traumatizing events. Statistical tests after cross-tabulations often lacked statistical power, yielding wide confidence intervals, and should be interpreted as tentative. The treatment-seeking crosstabulations notably represented small samples and lacked statistical power to detect some differences. The restriction of the study to a limited number of municipalities in the central coastal area and to people speaking a limited number of languages excluded a significant portion of the population from the sample. This suggests that specific surveys should be conducted among these populations, particularly the Amerindian and Bushinengue communities. Finally, this survey took place during the COVID-19 pandemic, which had significant social and mental health consequences, potentially affecting the observed prevalence rates.

Nevertheless, despite these potential limitations, the present study, resting on a robust methodology used in many different countries, provides the first estimates for the general population of French Guiana.

## Conclusion

Our initial hypothesis was that, given the prevalence of potentially traumatizing events within French Guiana and in the lives of immigrants fleeing disasters and violence, PTSD may be a greater problem than in mainland France. We show that the prevalence is 10 times greater, and that behind this, there are great differences between origins, sexes, and age groups. We also show that the majority of individuals do not care for their symptoms and that those who do often combine Western and traditional medicine. Integrating PTSD screening into primary care and community health programs, particularly in migrant and low-income areas, could reduce the treatment gap. Co-designed interventions that combine biomedical and culturally rooted practices may improve both reach and adherence.

## Data Availability

The data analyzed in this study is subject to the following licenses/restrictions: the data may be requested at the WHO collaborating center in Lille (Centre collaborateur de l’OMS pour la recherche et la formation en santé mentale – CCOMS, Service de l’EPSM Lille-Métropole, Lille, France). Requests to access these datasets should be directed to Deborah.SEBBANE@ghtpsy-npdc.fr.
