# Supplementary figures and images for: Post-traumatic stress disorder in French Guiana: prevalence and risk factors in the general population
Source: Front Public Health. 2025 Nov 26;13:1668105. doi: 10.3389/fpubh.2025.1668105 (PMC12689317; doi:10.3389/fpubh.2025.1668105)

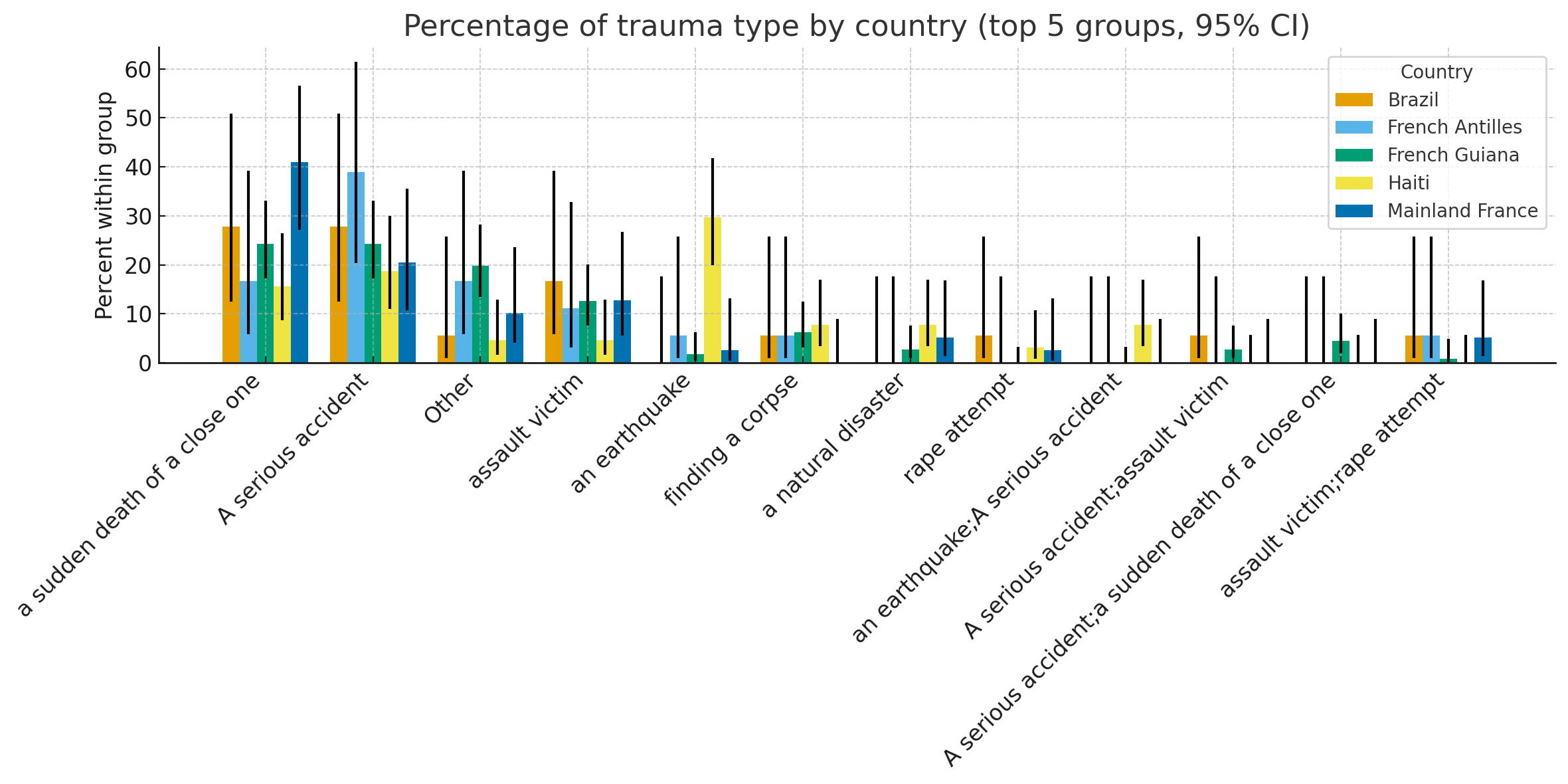

Supplement: Supplementary file 1 [file Image_1.png]

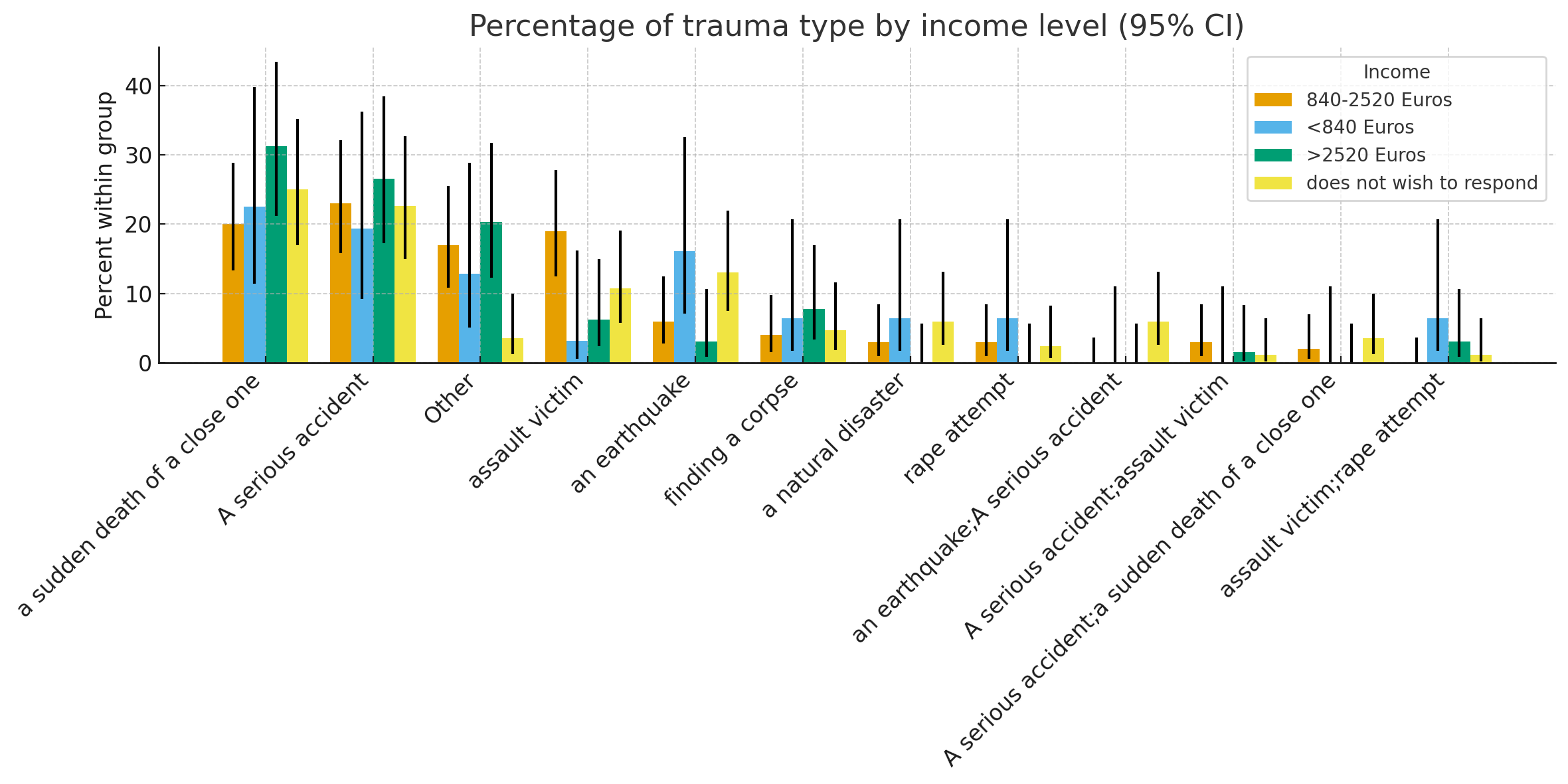

Supplement: Supplementary file 2 [file Image_2.png]

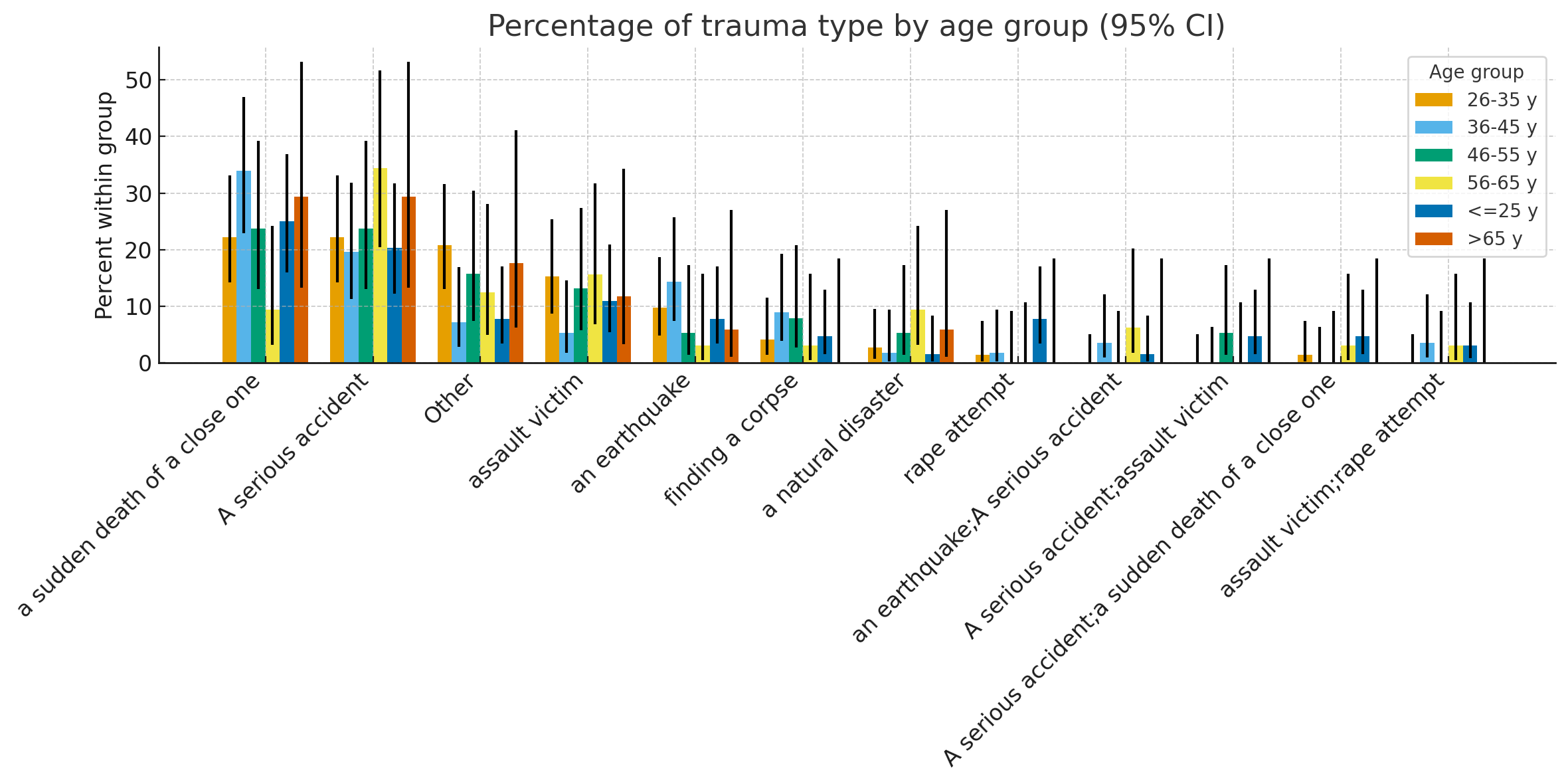

Supplement: Supplementary file 3 [file Image_3.png]

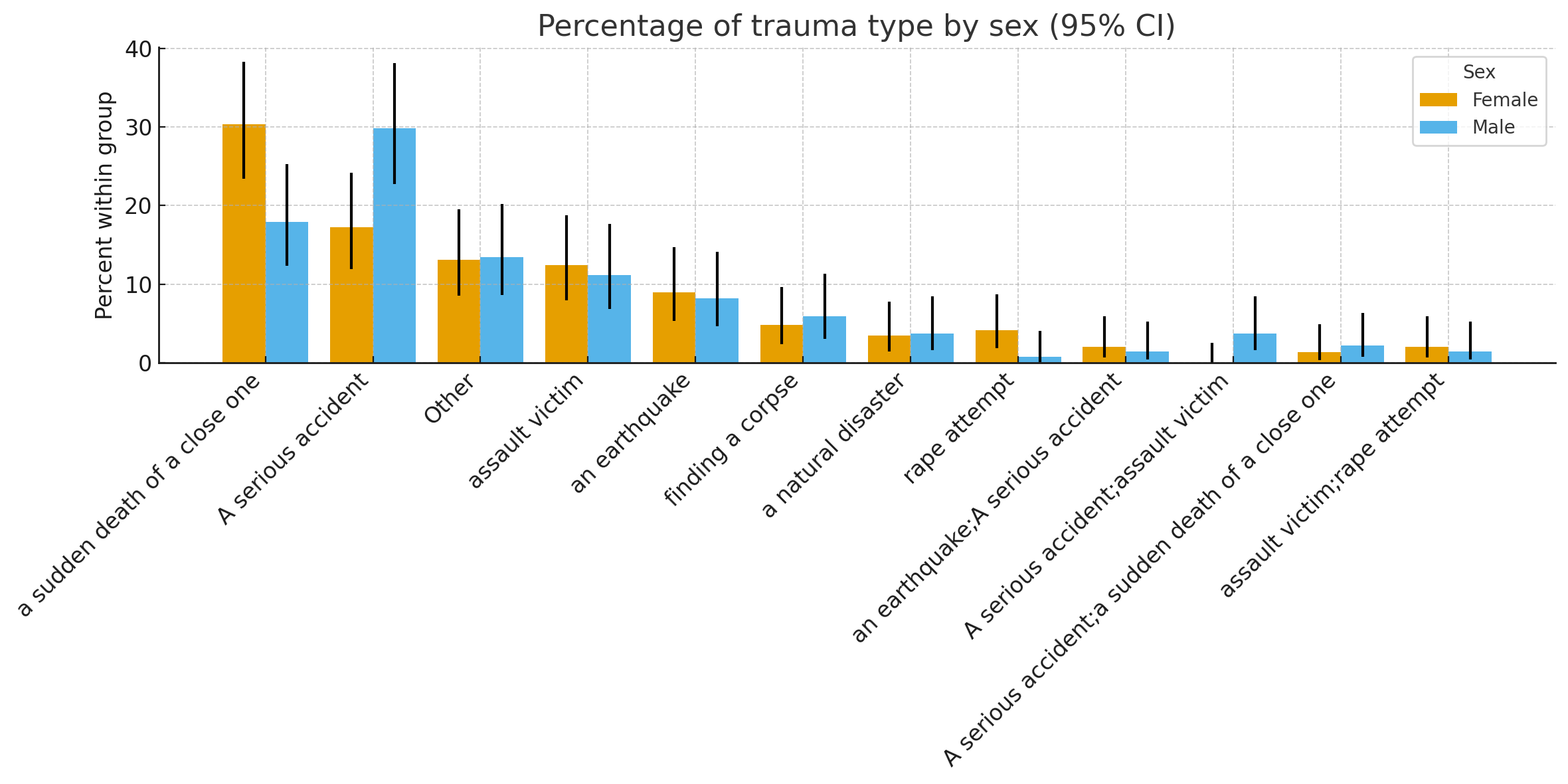

Supplement: Supplementary file 4 [file Image_4.png]
